# Self-Organized Criticality in Developing Neuronal Networks

**DOI:** 10.1371/journal.pcbi.1001013

**Published:** 2010-12-02

**Authors:** Christian Tetzlaff, Samora Okujeni, Ulrich Egert, Florentin Wörgötter, Markus Butz

**Affiliations:** 1 Bernstein Center for Computational Neuroscience, Institute of Physics III - Biophysics, Georg-August Universität, Göttingen, Germany; 2 Bernstein Center for Computational Neuroscience, Albert-Ludwigs Universität, Freiburg, Germany; 3 Neuroinformatics Group, Neuroscience Campus, VU Universiteit, Amsterdam, The Netherlands; University College London, United Kingdom

## Abstract

Recently evidence has accumulated that many neural networks exhibit self-organized criticality. In this state, activity is similar across temporal scales and this is beneficial with respect to information flow. If subcritical, activity can die out, if supercritical epileptiform patterns may occur. Little is known about how developing networks will reach and stabilize criticality. Here we monitor the development between 13 and 95 days in vitro (DIV) of cortical cell cultures (*n* = 20) and find four different phases, related to their morphological maturation: An initial low-activity state (≈19 DIV) is followed by a supercritical (≈20 DIV) and then a subcritical one (≈36 DIV) until the network finally reaches stable criticality (≈58 DIV). Using network modeling and mathematical analysis we describe the dynamics of the emergent connectivity in such developing systems. Based on physiological observations, the synaptic development in the model is determined by the drive of the neurons to adjust their connectivity for reaching on average firing rate homeostasis. We predict a specific time course for the maturation of inhibition, with strong onset and delayed pruning, and that total synaptic connectivity should be strongly linked to the relative levels of excitation and inhibition. These results demonstrate that the interplay between activity and connectivity guides developing networks into criticality suggesting that this may be a generic and stable state of many networks in vivo and in vitro.

## Author Summary

Learning depends crucially on the synaptic distribution in a neural network. Therefore, investigating the development from which a certain distribution emerges is crucial for our understanding of network function. Morphological development is controlled by many different parameters, most importantly: *neuronal activity*, *synapse formation*, *and the balance between excitation and inhibition*, but it is largely unknown how these parameters interact on different time scales and how they influence the developing network structure. In our work, we consider the well-known concept of self-organized criticality. We have measured how real cell cultures change their activity patterns during the first 60 days of development traversing through different stages of criticality. With a dynamic model we can reproduce the observed developmental states and predict specific time-courses for the network parameters. For example, the model predicts a delayed, overshooting onset of inhibition with a longer time to reach maturation as compared to excitation. Furthermore, we suggest that the balance of dendrites and axons in the mature state is quite sensitive to the initial conditions of development. These and several more predictions are accessible by future experimental work and can help us to better understand neuronal networks and their parameters during development and also in the mature state.

## Introduction

During the last years increasing evidence has accumulated that networks in the brain can exhibit “self-organized criticality” [1]–[3]. Self-organized criticality is one of the key concepts to describe the emergence of complexity in nature and has been found in many systems – ranging from the development of earthquakes [4] to nuclear chain reactions [5]. All these systems exhibit spatial and temporal distributions of cascades of events called avalanches which can be well described by power laws [6]–[8]. This indicates that the system is in a critical state [6], [9] and that similar dynamic behavior exists across many different scales. Several neural network models have predicted that neural activity might also been organized this way [10]–[14] and recently this had been confirmed experimentally [1], [15]–[17]. A recent study by Levina and colleagues [18] addresses the question how self-organized criticality can emerge in such networks in a robust way by using dynamical synapses, which alter their synaptic connection strength on a fast time scale. This contribution, which is able to analytically predict the network behavior, is a theoretical milestone in our understanding of criticality in neural systems. In general, however, theoretical and experimental investigations have so far usually focused on mature networks [1], [16] sometimes including adaptive processes [18]–[20]. Little is known how developing networks can reach a final state of self-organized criticality [10], [17], [21]. In the current paper, we are therefore experimentally investigating the different stages of developing cortical cell cultures [22] to assess under which conditions these networks develop into a critical state. Specifically we are asking the following questions: 1) do the investigated cell cultures undergo a significant transition in their activity states and how is this related to self-organized criticality and 2) can specific predictions be made with respect to network activity and connectivity which would explain the observed behavior. To address the second aspect we are designing a model to simulate network development, which is based on activity-dependent axonal and dendritic growth leading to homeostasis in neuronal activity [23]–[28].

## Results

### Experimental approach

**Figure 1:**
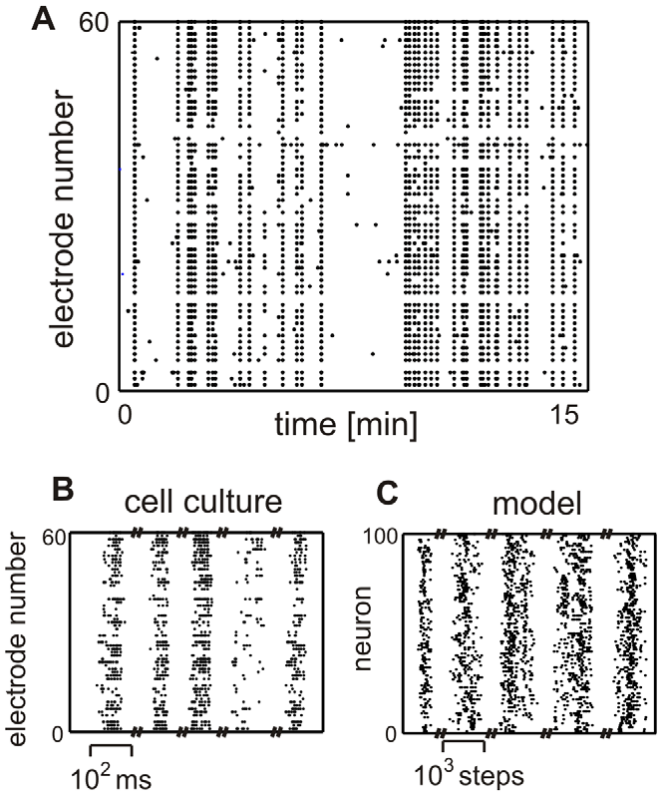
Raster plots at different temporal resolutions for experimental and model data. They are showing (**A**) the patterns of high burst-like activity and following pauses and (**B,C**) the activity patterns during some bursts. For graphical reasons, in panels (**B,C**) intervals between bursts have been shortened and do not correspond to the true intervals visible in panel (**A**). Thus scale bars refer only to the bursts.

In order to assess how self-organized criticality develops in cell cultures, we have monitored a total of 20 cultures and recorded their activity patterns between 13 and 95 days in vitro (DIV). In general, cultures start with about 500,000 dissociated cortical neurons, which develop over time into an interconnected network. To assess the different network states the activity at 59 electrodes was measured and analyzed at different DIV (see **Methods**). Figure 1 A shows 15 minutes of recorded activity for one typical culture at 42 DIV. At this temporal resolution individual bursts are visible as vertical dot-lines indicating activity at almost all electrodes, separated by rather long pauses which allow for robust separation of these bursts required for avalanche analysis. At a fine temporal resolution (Figure 1 B) one sees that the burst activity expresses certain patterns. Note, pauses have been graphically shortened in panels (B) and (C). Panel (C) shows the activity pattern that arises in our model, which at a first glance looks similar to that in the culture. Details about the model and an analysis which support similarity of model and real data, will be provided later. First we would like to describe the developmental stages in the cultures with respect to their avalanche distributions. In this work, avalanches are defined by the number of spikes between two windows without activity (see **Methods**).

**Figure 2:**
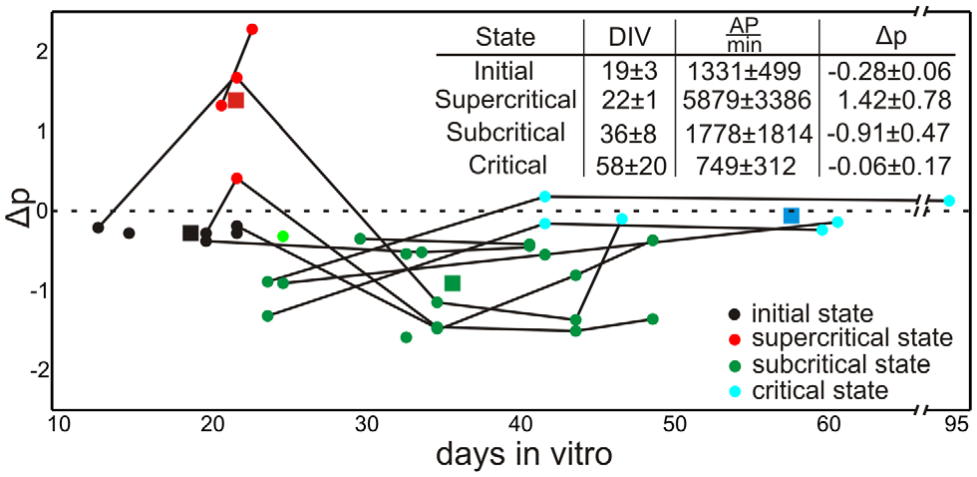
Development of the deviation from a power law 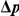 **of cell cultures.** The transitions from initial (black) to supercritical (red) to subcritical (green) and critical state (blue) can be clearly seen. Data from the same cell culture at different time points are connected. 14% of the total number of cultures has been tracked at 5 different time points, 7% at 4 time points, 29% at 3, 14% at 2, and 36% once. Squares indicate the mean values of DIV and 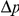 (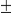 indicates the standard deviation), which are given in the inset Table, of the associated state. 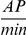 amount of action potentials per minute, therefore, mean activity.

**Figure 3:**
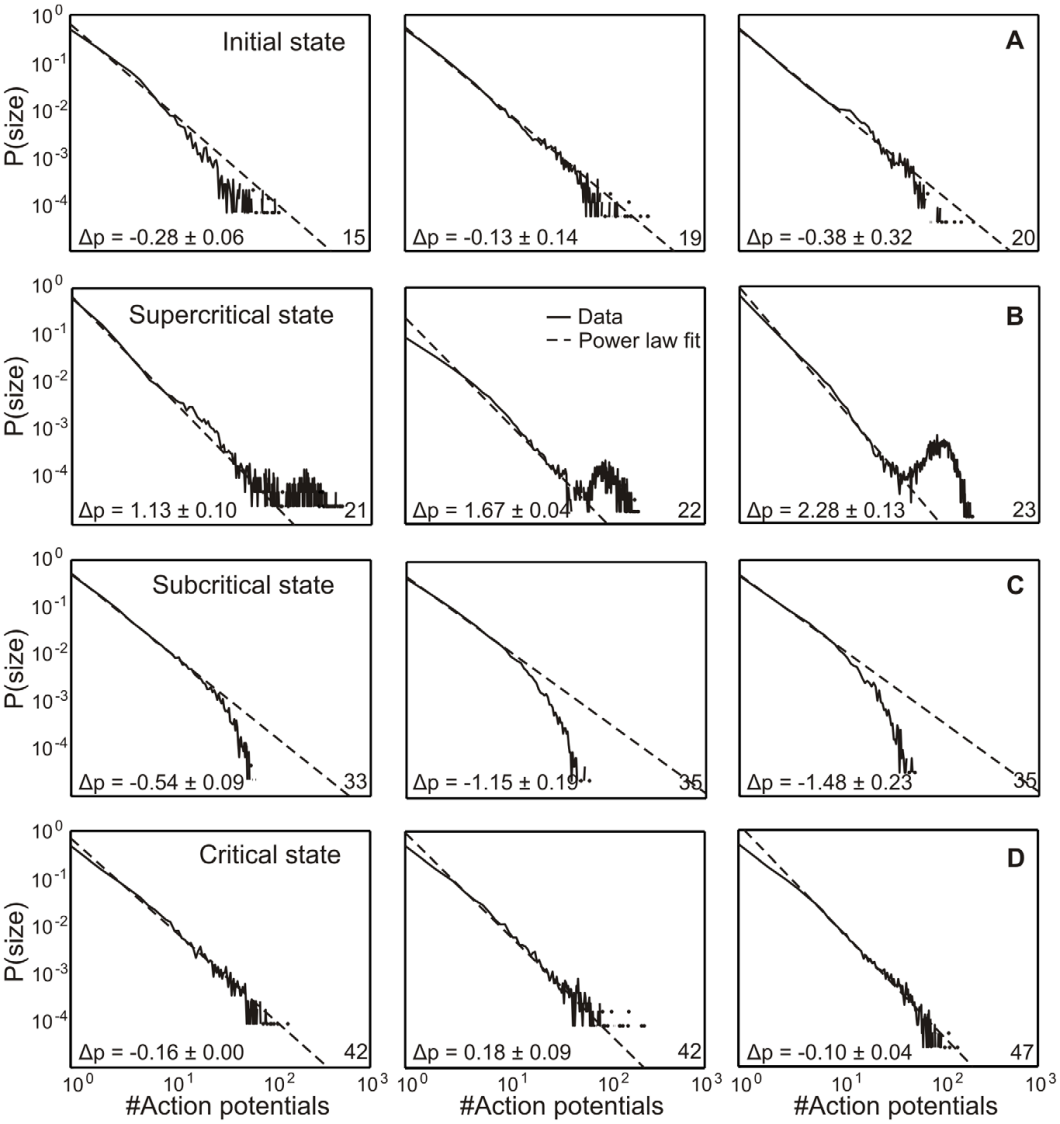
Avalanche distribution changes during morphological development of dissociated cell cultures. The dashed line indicates a perfect power law distribution. The deviation of the cell culture data from this line measures the criticality of these systems. For each state three different examples are shown. The age of each state of the cell cultures is given in the bottom right corner of the panels. (**A**) Initial state (on average 19 DIV); (**B**) supercritical state showing a “bump” of many long avalanches (on average 22 DIV); (**C**) subcritical state (on average 36 DIV) showing a depression and hence a lack of long avalanches; and (**D**) critical state (on average 58 DIV) with a good match to the power law line.

At early stages during development, usually before 13 DIV, connectivity is small and activity in the network very low. So, it is very difficult to obtain long enough recordings for plotting avalanche distributions. However, known from the literature [29], in this stage activity is best described by a Poisson like behavior. At about 13 DIV (see Figure 2), we receive the first distributions which develop towards criticality (Figure 3 A). Therefore, we call this state the *initial state*. The ideal power-law fit for each curve is shown by the dashed lines. If a distribution matches the power law line it can be called “critical” [6], [7]. A dominance of long avalanches is indicative of a supercritical state whereas a lack thereof is referred to as subcritical. This is measured by 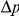, which gives the quality of fit between ideal power law and actual distribution. For a system in a supercritical state 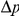 is larger and for a subcritical state smaller than zero (see **Methods**). Values of 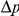 are also shown in the different panels of Figure 3. For the cultures, we receive at (on average) 19 DIV values of 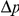 in the interval from 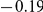 to 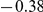. While this shows that the system develops towards criticality, we also observed that this behavior is very unstable. Quickly, within just (on average) one/two days, the distributions change shape and develop a substantial “bump” for larger avalanches. This indicates that at (on average) 22 DIV the network enters a supercritical regime (Figure 3 B). After (on average) 36 DIV network activity is curbed and it reaches a subcritical regime (Figure 3 C). This can be seen by the decrease of the distribution at larger avalanches. At (on average) 58 DIV the system becomes finally critical (Figure 3 d). Here we find that the deviation from a power law is nearly zero (for these examples 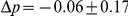). In general we find that the differences between all states are significant for the measured values of 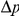 (ANOVA test). Figure 2 provides the data of all 20 cultures (see **Methods**) divided into the different states. All completely measured cultures undergo the same transitions from initial (black) to supercritical (red) to subcritical (green) and finally to a critical state (blue). The overlap between the first two states results from the very quick transition between them together with small differences in the speed of development of the different cultures. Average values of 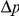 for these four states are 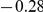, 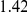, 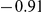, and 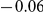 (see Table in Figure 2). Differences are significant using the multiple comparison procedure with Bonferroni correction based on the one-way ANOVA test. Only the difference between the initial and critical state is not significant as in the initial state the network develops towards criticality until strong morphological changes set in (see **Phase I**). However, the activity given by the number of action potentials per minute is for the supercritical state significantly higher than for the initial, subcritical and critical state, which has the lowest mean activity. These were the only differences that were observed.

In summary, these results show that there is a characteristic time course in the development of the avalanche distributions. The system starts with low activity and then enters a transitory initial state. Quickly it leaves this state and, passing supercritical and subcritical regimes, reaches the critical state.

### Modelling approach

#### Neurite growth and retraction towards firing rate homeostasis

The model uses two opposing mechanisms of axonal and dendritic growth and is driven by the goal to reach homeostasis of the mean firing rate. The first mechanism regulates dendritic growth probabilities inversely to neuronal activity and the second is the axonal outgrowth promoted by activity. Specific choices for the model are being discussed in the Discussion section, where we also summarize the different specific predictions made by the model and described in detail in the next sections.

As will be shown below, the model is capable of reproducing all different patterns of neuronal activity (Figure 3) based on the implemented rules for activity-dependent structural network formation. A neuron is represented by its membrane potential 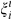 and its inner calcium concentration 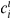 (see **Methods**) at the time point 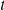. After a disturbance, these variables will decay in time to the resting values 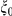 and 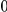 for the membrane potential and the calcium concentration, respectively. Every time a neuron generates an action potential (see Equation 15 in **Methods**), its calcium concentration increases by a constant 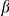.

Dependent on the difference between the current calcium concentration and a desired homeostatic value 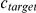, the neuron changes its input (dendritic acceptance 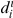) and output (axonal supplies 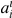) by ways of a simulated growth or withdrawal process (see **Methods**). The intersection between input and output of two neurons 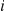 and 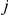 determines the synaptic density 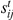, and hence the connectivity, between them.

The difference between an inhibitory and excitatory neuron is defined by constants 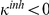 and 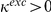, which are prefactors of 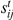.

**Figure 4:**
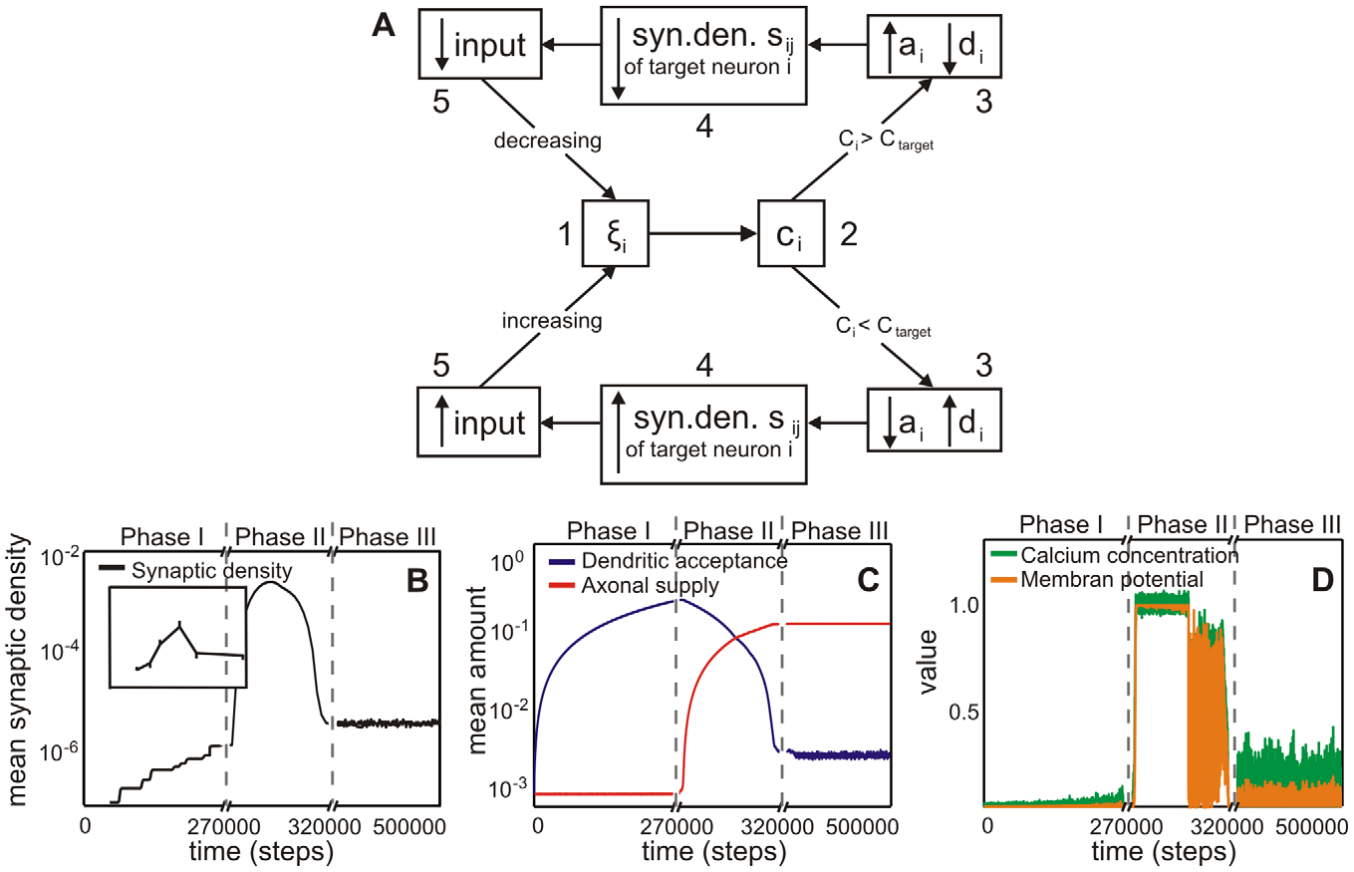
The development of the model shows three different phases (Phase I, II and III). (**A**) Box diagram of the model feedback loop. Variables are membrane potential 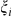, calcium concentration 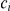, axonal supply 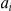 and dendritic acceptance 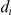, and connectivity 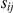 between dendrite 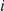 and axon 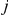 and the constant homeostatic value 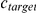. The up and down arrows indicate if a variable is increased/decreased. For details see main text. (**B**) The mean synaptic density 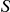 develops comparable to experimental findings in cell cultures (see inset from [24]). Note, time axis has been stretched in the middle. (**C**) Development of the average axonal supplies and dendritic acceptances. The network model passes through three different developmental phases: the first phase is characterized by a pronounced increase of the dendritic acceptance. During development, the network undergoes a transition (second phase) and finally it reaches a homeostatic equilibrium (third phase) with more axonal supplies than dendritic acceptances. (**D**) Network activity and calcium concentration change accordingly. At the beginning, activity rises slowly until a transition happens. During the transition, activity reaches its maximum and subsequently decreases to a homeostatic value.

In summary, the model comprises a negative feedback loop of the following kind (Figure 4 A): Neuronal activity (1) determines the calcium level (2) in the cell. This level leads to the simulated growth pattern of the neuron(s). The growth pattern determines the effective amount of axonal supplies and dendritic acceptances (3). Thus, growth of many neurons, influencing their respective neuritic offers, will lead to different synaptic densities (4) between neurons. We use this synaptic density as the simplest way to estimate the inputs (5) to any given cell. This input will then determine the cell's activity closing the loop at (1).

These interactions lead to the effect that the model development passes through three different morphological phases (Figure 4 B–D), which we will first describe qualitatively and in the following subsections also analyze mathematically as far as possible.

The initial supplies of the axons and acceptances of the dendrites are chosen such that no connections exist. As a consequence of the resulting too low activity the dendritic acceptance increases to build synapses and to enhance the activity in the first developmental phase I. It rises slowly and, at a certain point in time, increases explosively towards a maximum. Parallel to this increase in activity, the system undergoes a morphological transition (Phase II) until it reaches homeostasis (Phase III). As discussed later (see **Discussion**), this is similar to the morphological development in such cultures (see inset in Figure 4 B). At the final stage the mean activity is equal to the homeostatic value (see **Methods**) and changes of the axonal supplies and dendritic acceptances are negligible.

The three different phases in the above described development can be largely understood in an analytical way and we can also describe to what degree the system approaches criticality. The difficult recurrent processes, which drive the interactions within a network and lead to a specific avalanche distribution, however, defy analytical analysis and can only be obtained from simulations. Additionally, the effects of inhibition on the network dynamic in the different developmental phases are tested by simulations.

### Phase I: First developmental phase 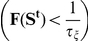


The first phase (Phase I) of the network development is characterized by dendritic growth to establish first synaptic contacts and to rise neuronal activities. At the beginning of the model development the dendritic acceptance increases (Figure 4 C). By this outgrowth the system creates synapses and forms a network. The distribution of the avalanches, the mean membrane potential 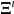, and the mean calcium concentration 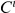 also changes (mean values over all neurons are given as upper case letters, while lower case letters indicate individual values). Similar to real cell cultures, all neurons at this phase are excitatory. With the help of a *mean field approach* it is possible to calculate average membrane potential 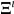 and average calcium concentration 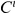 during this phase. Different from real networks, where the activity is too small to render reliable measurements for very early developmental stages, in the model we can also analyze these. For this, the term 
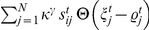
, which determines the increase of the membrane potential 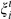 according to the activity of the connected neurons 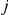 in Equation 14 (see **Methods**), is simplified to a product of the mean membrane potential 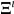 and an monotonous function dependent on the mean synaptic density 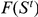 (see below) and we get for the activity change:
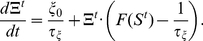
(1)The differential equation of the calcium concentration (Equation 15 in **Methods**) can be written as:
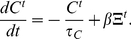
(2)With these equations, we can now consider three different degrees of synaptic densities in the first phase 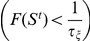; namely zero, small, and medium densities and for Phase II 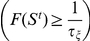 with a large density.

#### Network development before synapse formation 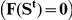


For the initial conditions of the model without connectivity, 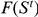 is set to zero. Therefore, from Equation 1 one can obtain that the mean activity 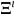 reaches the resting potential:
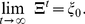
(3)If this solution is entered in Equation 2, we get:
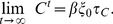
(4)Thus, also the mean calcium concentration reaches a constant value dependent on 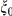.

Taking the limit 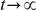 corresponds to letting the system under the given condition 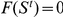 relax into its end state. Note however, that the actually ongoing development (Figure 4 A) will curtail this condition as eventually 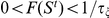.

**Figure 5:**
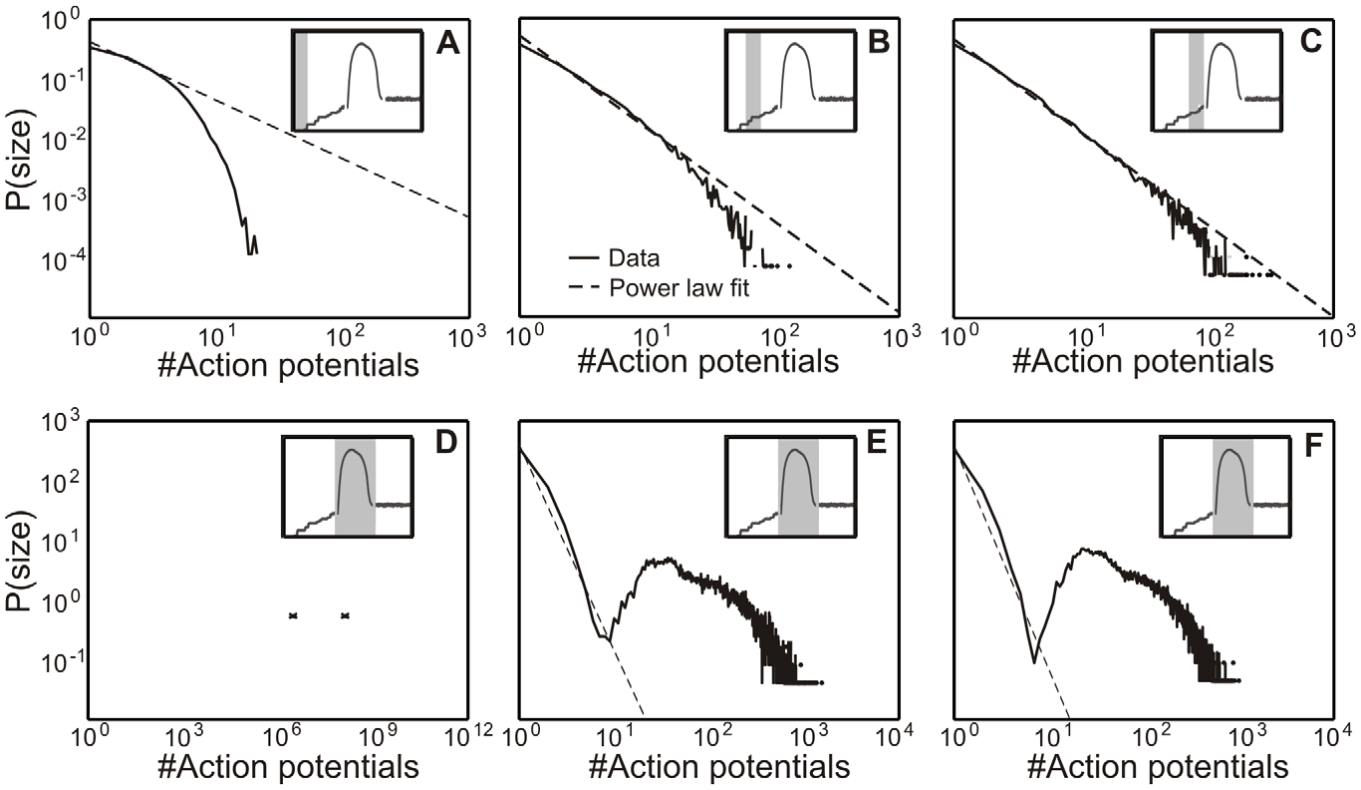
Avalanche distribution of the model in Phase I and II. Gray areas in insets (taken from Figure 4 B) show the time point in the development. **(top):** (**A**) Initially, the connectivity between neurons is zero. Because of that a Poisson-like distribution describes the spontaneous neuronal activity best. (**B,C**) With increasing 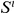 (B: 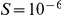; C: 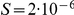), the avalanche distribution turns from a Poisson into a power-law like distribution similar to Figure 3 A. **(bottom):** In Phase II without inhibition (**D**), no real avalanche distribution can be observed and one sees only one or two “avalanches” (marked by a cross). Adding inhibition brings the system back into a stable, albeit supercritical regime. Within a wide tested range (Table 2), the amount of inhibition does not significantly change the degree of supercriticality. (**E**) Network with weak inhibition 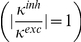 and (**F**) with strong inhibition 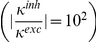.

From Figure 5 A we can see that the avalanche distribution shows a poissonian form. This is also reflected by a large negative value for 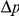 (Table 1, first row). This changes as soon as the model begins to make the first connections between neurons as shown in the following.

**Table 1:** The mean synaptic density 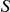 influences the membrane potential 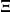, avalanche distribution, and mean firing rate per time step 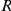.

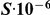	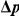	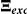	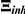	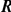
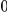	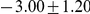	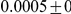	-	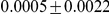
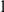	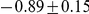	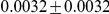	-	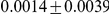
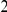	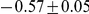	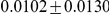	-	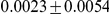

With rising density the activity increases and the distribution develops from a Poisson to a power law like form. 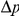 = value for the deviation from a power law, 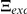, 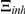 mean membrane potential for excitatory and inhibitory neurons. Note, inhibition is not yet present in this phase.

#### Network development with small and medium connectivity 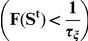


As soon as the system has reached small connectivity, the behavior of the membrane potential, calcium concentration, and avalanche distribution changes. This corresponds to a situation where we have 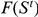 larger than zero but smaller than 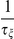. So, the system is still in Phase I. It is easy to see that the dynamics change again if the density function becomes larger than 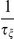 and this is later discussed in Phase II.

We can solve the differential equations (Equation 14 and 15) for the mean variables with standard methods and get:
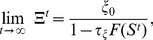
(5)

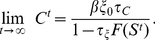
(6)As the synaptic density function 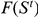 is in this case smaller than 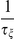, the product 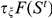 is between zero and one (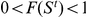). Therefore, the solutions for 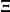 and 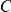 with connectivity (Equations 5 and 6) are larger than without (Equations 3 and 4) but remain bounded. As a consequence, the membrane potential and the rate 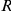 in the system rise slowly in time (Figure 4 B–D, Phase I) dependent on the density which still growths as homeostasis is not yet reached (Figure 4 B).

Also the avalanche distribution changes slowly with rising connectivity and activity from a Poisson to a power law distribution (see transition in Figure 5 A–C, Table 1).

In the whole Phase I, the network never attains steady state. Hence connectivity and activity continue to change. Criticality essentially follows these changes. The transition from small to medium synaptic density only leads to a qualitative change in the distribution (Figure 5 B,C), which now becomes very similar to the ones measured around 18 DIV in the real cell cultures (Figure 3 A).

### Phase II: Second developmental phase 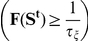


Phase II of the network development is characterized by an overshoot in network activity. The membrane potential and calcium concentration (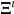 and 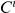) reach their maximum. This causes a phase transition in axonal and dendritic development: At that point, the dendritic acceptance begins to shrink and the axonal supply increases (see 4 C,D, Phase II). Moreover, during such transitions (accompanied with the formation of very many synapses) the action of the transmitter GABA switches from excitatory to inhibitory due to a change in the intracellular chloride concentration [30]. As we do not model changes in ion concentrations, we just change 20% of all neurons and assign them a negative value of 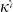, thereby making them inhibitory (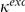 is changed to 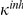 in this second phase). To determine the influence of different degrees of inhibition, the ratio of 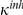 to 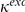 is chosen differently in different experiments (Figure 5 D–F).

We can calculate the membrane potential as before with Equations 1 and 2 now with the constraint 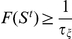 for a network without inhibition. As the membrane potential has by definition an upper limit of 1, the limit for 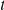 to infinity during the phase transition (Phase II) is:
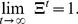
(7)The calcium concentration has no upper limit and will theoretical rise to infinity
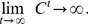
(8)As the system remains only for a finite time in this second stage, 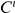 will, however, remain finite. The mean membrane potential on the other hand reaches in the simulations indeed a value of 1 while the calcium concentration approaches 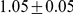 (Figure 4 D).

If the membrane potential is close to one, neurons theoretically fire at every time step. Due to the given refractory period of 4 time steps, however, only 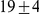 out of 100 neurons fire on average in one time step. Without inhibition too many neurons are active during this stage and distributions cannot be reasonably assessed because one will only measure one or two “endless” avalanches (Figure 5 D).

Introducing inhibition changes this behavior substantially. The mean membrane potential decreases from 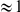 to 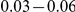 (Table 2) and the avalanche distribution shows now a measurable supercritical behavior (Figure 5 E,F). For measuring this avalanche distribution both excitatory and inhibitory neurons are considered. The membrane potential for the inhibitory neurons 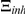 is larger than that for the excitatory neurons 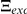. This is due to their lower density (20% inhibitory as compared to 80% excitatory neurons).

**Table 2:** The system is overly active without inhibition so that it is not possible to determine the avalanche statistics (there exist one or two large avalanches across the whole second phase).

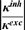	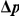	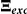	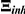	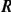
0	-	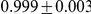	-	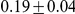
1	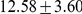	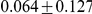	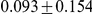	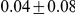
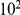	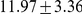	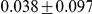	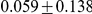	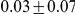
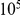	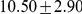	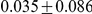	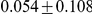	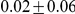

Inhibition strongly dampens this and one arrives at supercritical behavior. 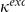, 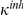 weight of the excitatory and inhibitory connections. All other variables as in Table 1, above.

As in Phase I, the network will not reach a steady state in Phase II, either. However, by contrast to the first phase where activity and connectivity is slowly growing, in the second phase, connectivity and activity is quickly getting overly strong (Figure 4 B–D, Phase I and II). Therefore, the system remains supercritical for the whole second phase until pruning is reducing connectivity to the homeostatic value in Phase III. Note, that stronger inhibition dampens the membrane potential and the firing rate considerably but does not influence the supercritical behavior of the system; 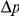 (Table 2) remains essentially the same across five orders of magnitude of increased inhibition (see also Figure 5 E,F).

### Phase III: Third developmental phase

#### Firing rates become independent from parameter settings

Phase III is that of morphological homeostasis of the network and the network has now equilibrated reaching a steady state, where firing rate is stable in the mean.

It is obvious that the average steady state rate 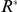 (the asterisk 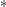 indicates steady state values) follows the averages of potential 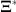 (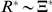) and synaptic density 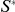 (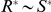), while it is inversely related to inhibition 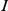 (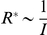).

Let us first consider the system without inhibition. Also in this case in Phase III we receive a stable rate with constant 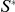. As a consequence 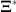 should be constant, too. The top row for each fixed point (FP) 1–3 in Table 3 demonstrates that this is indeed the case. (The meaning of the different fixed points will be discussed in the next section. This can for now still be ignored.)

**Table 3:** In the homeostatic state (Phase III), the membrane potential 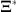 is independent of the attained fixed point (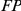) and the inhibition (ratio of 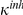 to 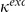).

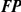	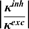	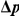	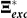	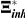	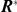	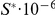
**1**	0	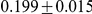	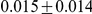	-	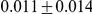	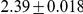
****	1	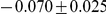	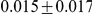	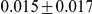	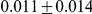	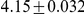
	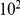	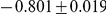	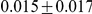	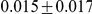	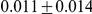	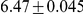
	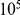	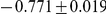	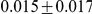	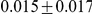	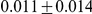	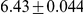
**2**	0	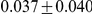	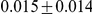	-	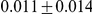	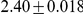
	1	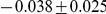	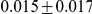	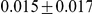	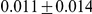	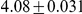
	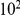	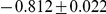	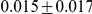	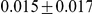	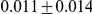	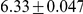
	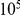	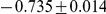	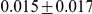	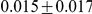	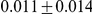	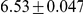
**3**	0	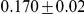	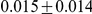	-	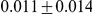	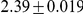
	1	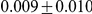	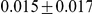	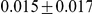	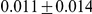	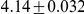
	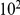	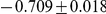	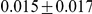	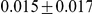	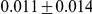	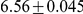
	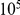	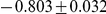	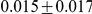	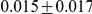	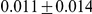	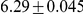

By contrast, connectivity 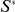 and the avalanche distribution 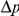 changes with the level of inhibition.

With different levels of inhibition the steady state connectivity 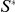 changes. Larger inhibition leads to larger connectivity and vice versa. This is due to the effect that inhibition tries to lower the rate. As a consequence of the system being homeostatic (Equations 14–17) connectivity will increase to keep the rate constant. Because of the constant rate and the co-variation of inhibition and connectivity, we expect again that the membrane potential 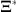 should be constant. Table 3 shows this, too. For each ratio of 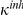 and 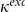 the membrane potential and number of spikes (firing rate 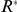) remain the same.

As a central conclusion we observe that rates 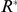 and membrane potentials 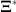 are in Phase III fully invariant against system parameters and initial conditions. Connectivity 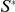, however, is influenced by the level of inhibition.

#### Analytical approximation of the firing rate in the steady state

As the firing rate is the most accessible variable in cell cultures, we are now showing how to compute the firing rate in the model analytically. When the network is in a homeostatic equilibrium, the calcium concentration for each neuron on average equals the target value 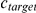. With this, and assuming that action potentials are uniformly distributed in time (see Supporting Information **Text S1**), it is possible to calculate the firing rate 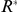:
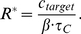
(9)This solution quite accurately approximates the values for 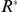 obtained by the simulation (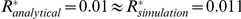 see Table 3). A more detailed analysis shows that the remaining small difference arises from the discrete sampling in the numerics (not shown).

#### Homeostasis criticality is influenced by inhibition

**Figure 6:**
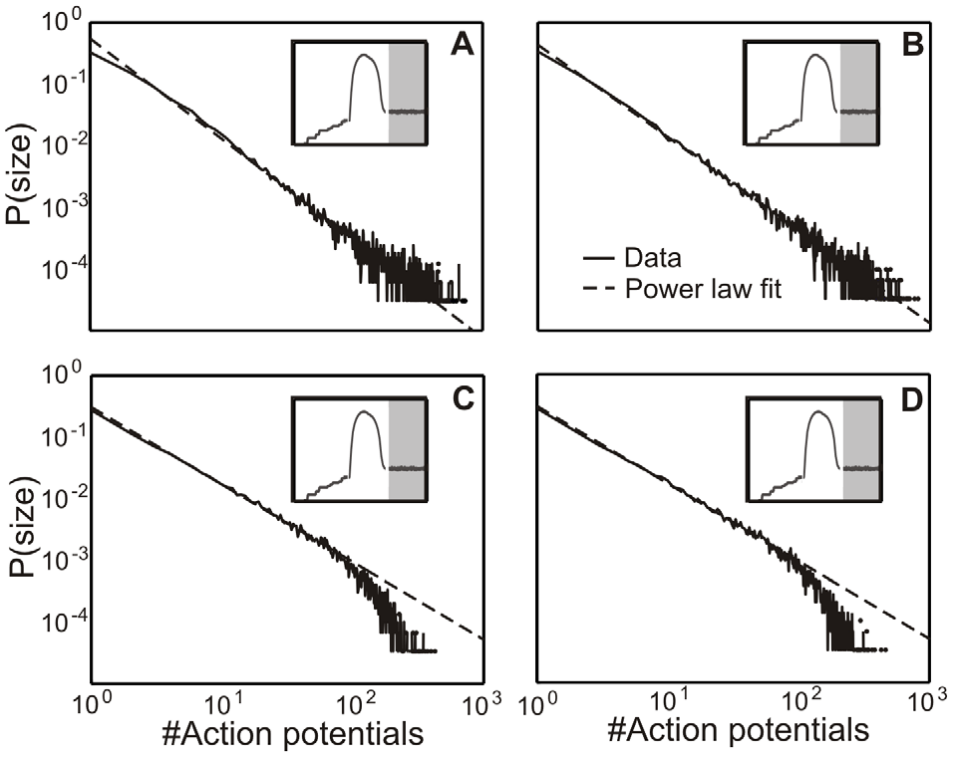
In the homeostatic equilibrium (Phase III), the degree of inhibition determines whether the network finally reaches a critical state or remains sub- or supercritical. As a characteristic example the avalanche distributions from fixed point 1 (see Table 3) are shown. (**A**) A purely excitatory network stays slightly supercritical although network activities are homeostatically balanced (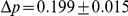). (**B**) If the absolute value of the inhibitory strength 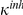 equals the excitatory strength 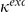 the network becomes critical (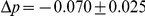). Here the total number of inhibitory synapses is about 20%. (**C–D**) Higher levels of inhibition (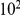 for C and 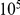 for D) keep the network in a subcritical regime (C: 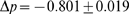; D: 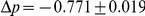).

Above we observed that inhibition influences the final connectivity that gives rise to network homeostasis. Here we find that also the avalanche distribution is dependent on inhibtion (Figure 6). Without inhibitory neurons the distribution is slightly supercritical. With 20% inhibitory neurons with the same synaptic weighting as the excitatory neurons (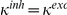), we obtain a critical distribution. Further increase of the inhibition to a ratio 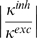 of 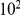 drives the system significantly into a subcritical regime. A further increase to 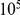 does not significantly increase subcriticality anymore.

This demonstrates that criticality after equilibration, hence on the long run, depends on connectivity but neither on the mean membrane potential 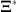 nor on the resulting average firing rate 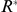.

#### Criticality is subject to acute changes in inhibition

**Figure 7:**
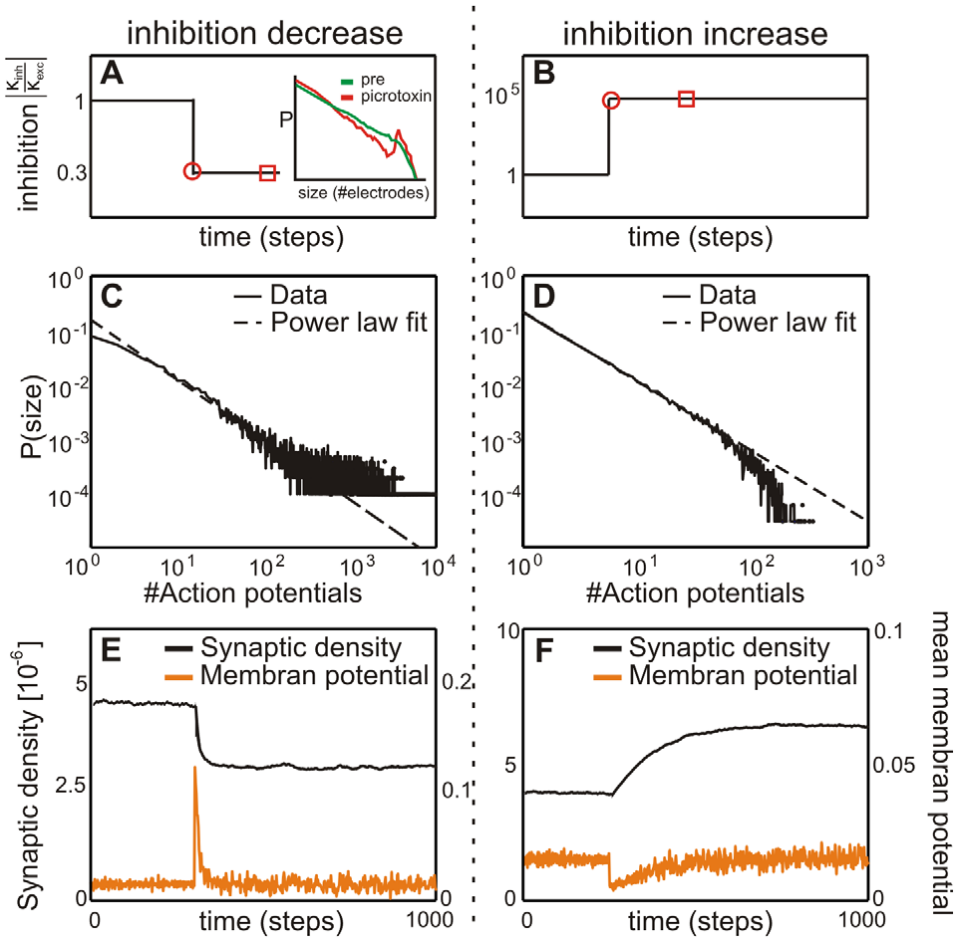
A sudden change of the inhibition in Phase III destabilizes the system. Inhibition is suddenly decreased/increased (**left/right column**) as shown in panels (**A**) and (**B**). After the jump, the avalanche distribution becomes (**C**) supercritical (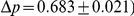 or (**D**) subcritical (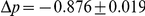), respectively. Distributions were plotted at the time point marked by the open disks in A and B. The reduced inhibition case is well backed-up by experimental data [1] as a similar change in criticality was observed in mature cell cultures after artificially increasing inhibition (compare inset). After some time (open square marker) distributions change and are then those shown in panels A and D in Figure 6. Now we have in both cases somewhat reduced (absolute) 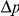 values as compared to those directly after the jump (now 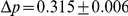 for the supercritical case Figure 6 A and 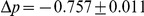 for the subcritical case Figure 6 D). Note, however, that we *do not get back to the initial criticality* (Figure 6 B, 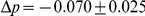). Parallel to this, the bottom panels (**E,F**) show that in both cases connectivity remains also changed. Activity, on the other hand, fully builds back.

This can be nicely demonstrated by disturbing an equilibrated system by a sudden change of inhibition (Figure 7). After such a jump, connectivity 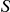 (and, thus, criticality, see Figure 7) changes, but mean membrane potential 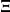 and rate 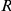 will relax back to their previous values. This long term process is initiated by the activity change that follows the artificially induced change of inhibition. Panels C and D in Figure 7 show that immediately after the jump, criticality changes to relatively high(low) values for 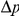 for the super(sub)-critical case (left vs right columns in Figure 7). A similar experiment has been performed by Beggs and Plenz [1] (see inset in Figure 7 A) where reduced inhibition also led to a supercritical system. While in our system activity fully builds back, super(sub)-criticality does not.

A comparison between panel B in Figure 6, which represents the fully relaxed case, with panels C and D in Figure 7, which represent the situation immediately after the jump, shows this clearly. Hence, while the activity change leads to an immediate change in criticality, it is the lasting change of connectivity that leads to the fact that also the changed criticality persists albeit on a reduced level.

Thus, the model predicts that sudden activity changes should affect criticality in Phase III, but in a reversible way. Lasting changes of inhibition, on the other hand, should also lead to lasting small changes in the criticality *without* affecting the mean firing rate in the network.

### Dynamic network behaviour: Isoclines and fixed points

**Figure 8:**
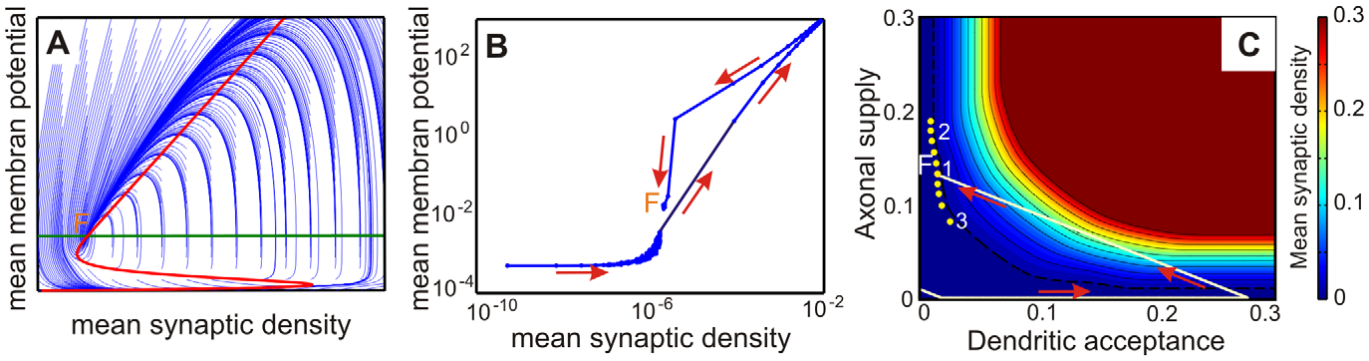
Development of the network in phase space. (**A**) Here the hysteresis curve of the mean membrane potential 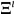 against the mean connectivity 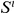 described by Equation 10 is displayed together with its possible trajectories (blue). 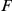 marks the equilibrium or stable point of the network. (**B**) Hysteresis curve from the simulation. (**C**) Different representation, which shows that the equilibrium 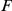 represents a region of fixed points with approximately equal connectivity. The axes represent here axonal supply and dendritic acceptance. Color indicates the calculated average connectivity 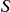. Depending on the initial state, the model grows into a fixed point of an omega limit set (yellow circles, region 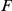) lying on a hyperbola (dashed line), thus with approximately equal connectivity 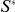. The “bumpy” shape of the hyperbola is due to grid aliasing effects.

So far we have described the three development phases for our network model showing how criticality depends on network state, where the final state suggests some kind of fixed point behavior. In the following we will assess to what degree this process is characteristic for the system. To this end, we calculate its nullclines analytically [24] and compare these results with the simulations in Figure 8. For simplicity here we treat only a purely excitatory network.

To be able to solve the problem analytically we assume that the change of the connectivity 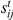 between neurons and their membrane potential 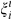 is slow and derivatives can, thus, be set to zero. Furthermore, on longer time scales the differences between neurons are negligible and only the behavior of the means need to be considered. As a result one can calculate the nullcline of this system (see Supporting Material **Text S2**), which describes a hysteresis curve (Figure 8 A):
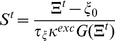
(10)

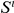
 and 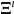 are the mean values of 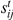 and 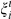 over all neurons. 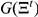 is a sigmoidal function as an approximation for the Heaviside function 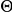, which determines when an action potential is generated (see Supporting Material **Text S2**). In Figure 8 A we also plot the trajectories which belongs to this system and the other (trivial) nullcline 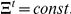, which describes the fact that the system develops into homeostasis. At fixed point 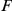 development stops. In Figure 8 B we plot the actual development of 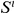 and 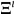 observed in the simulations. Ideally this curve should match one of the trajectories in panel A and one can see that this is essentially the case. The main deviation arises from the fact that, due to the required simplifications, the analytical solution in panel A shows during the phase transition (Phase II) infinite growth and this cannot be achieved in the simulation. This leads to a reduction in the rising slope of panel B and to the fact that the fixed point 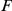 is shifted closer to the inflexion point of the isocline.

When considering axons and dendrites separately, fixed point 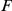 splits into a zone of many points, which correspond to the same connectivity 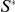, and hence lie on a hyperbola in Figure 8 C (dashed line). These fixed points form an omega-limit set in phase space and are represented by the equilibrium point 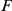 in the 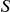-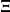-space. The approximate path of a trajectory from panels A and B is shown in Figure 8 C by the solid white line. Above we had stated that rates 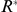 and membrane potentials 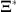 are in Phase III fully invariant against system parameters and initial conditions, while connectivity 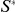 is influenced by the level of inhibition. To this we can now add that the actual balance between axonal supply 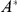 and dendritic acceptance 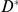 (location of the different fixed points) remains dependent on the initial conditions (as well as on the inhibition) and should, therefore, be the most sensitive developmental parameter, e.g. much susceptible to pharmacological interference.

Furthermore, as the rate essentially follows 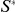 and 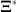 and inversely 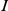, we can state that the isocline in Figure 8 A will, for larger inhibition, be shifted diagonally upwards away from the origin shifting the fixed point to a higher synaptic density.

The dynamic behavior shown in Figure 8 is similar to that observed in the studies of Van Ooyen and Van Pelt [24] and our results show that the three development phases (Phase I, II and III) of this system are generic and independent of the chosen simulation parameters and confirm the existence of a strong phase transition.

### Comparison between cell culture and model development

**Figure 9:**
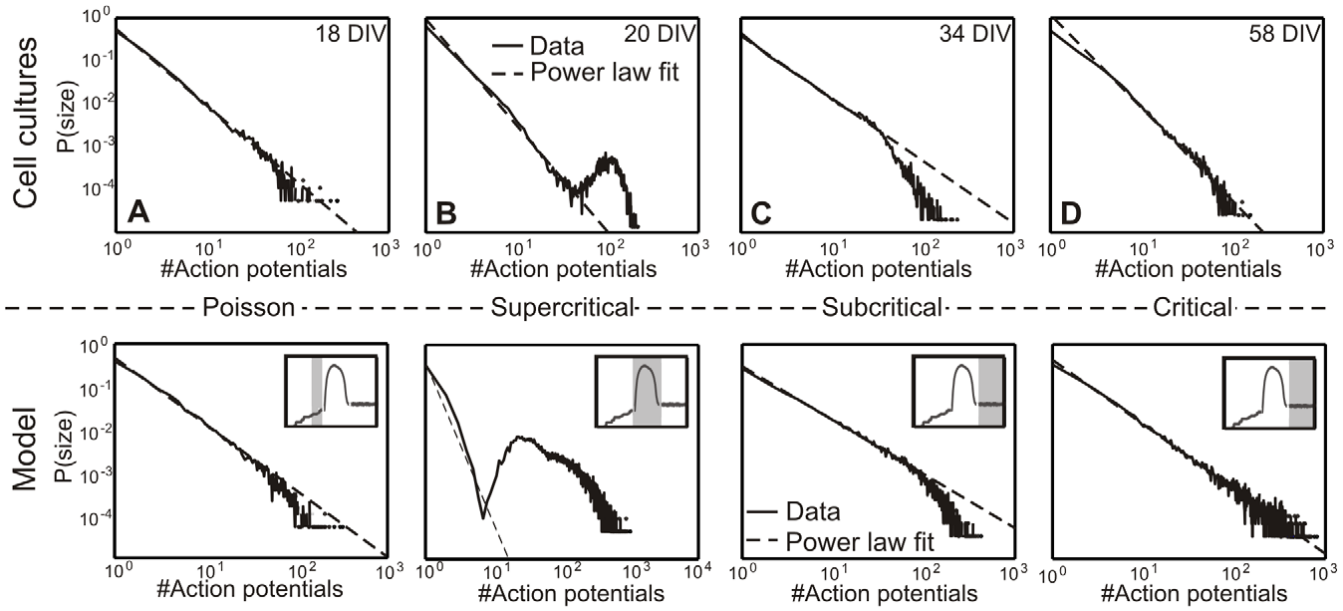
Comparing real data (top) with model (bottom). (**A**) Initial phase, (**B**) supercritical phase, (**C**) subcritical phase, and (**D**) critical phase.

Figure 9 shows a comparison of the different criticality states between cell culture (top) and model data (bottom) summarizing some of the observations from above. Additionally, the exponent of the avalanche distribution and the time bins 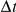 are given in Table 4 for each state in model and cell cultures. In the model, at the end of the transition from Poisson to power law (Figure 5 C), little connectivity in Phase I leads to an initial state similar to that observable in dissociated cell cultures (Figure 9 A). This is followed by strongly rising synaptic density in Phase II (B). Accompanying the overshoot in network activity and connectivity, the model network passes a transient phase of supercriticality (B, bottom) as do the cell cultures (B, top). Depending on the chosen strength of inhibition, we obtain in Phase III a subcritical state for the model (C, bottom, 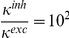) similar to that in cell culture data (C, top). Thereafter, still in Phase III, we have gradually reduced the inhibitory strength to 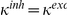, hence balancing synaptic strength for inhibition and excitation (while keeping the number of inhibitory neurons constant). This leads to a final critical state in the model (D, bottom) similar to that found in cell culture data (D, top).

**Table 4:** The exponent of the avalanche distributions and the time bins 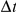 are given for the model (top) and cell culture (bottom) data in the different states.

Phase	parameter	initial	supercritical	subcritical	critical
Model	exponent	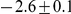	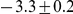	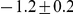	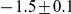
	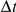 [steps]	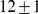	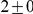	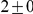	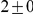
Experiment	exponent	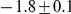	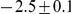	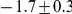	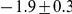
	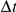 [ms]	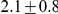	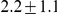	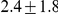	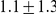

Thus, this predicts that that developing inhibition is an important factor for the course of criticality in developing neuronal networks. Only if inhibition in the model is lowered in Phase III again, the network becomes critical. Therefore, it is likely that overall synaptic pruning in developing networks not only affects excitatory but also inhibitory synapses [31]. Moreover, neuronal networks seem to reach firing rate homeostasis earlier than the equilibrium for maturing inhibition (compare discussion in Figure 7).

Additional inter-spike interval (ISI) and cross-correlation (CC) analyzes have been performed. ISIs and CCs are very similar between cultures and model across all stages but they do not contain interesting features (like oscillations) and therefore we do not show these diagrams here to save some space.

### Predictions of the model

The following predictions are derived from the model:

- Criticality at the end of development is optimally reached with 20% inhibition with a strength equal to that of the excitation. This observation does not depend crucially on the distribution of the inhibitory neurons and corresponds approximately to the normal degree of inhibition in cortical networks and cultured networks, respectively. A higher degree leads to sub- and a lower degree to supercritical behavior. A subcritical state is observed in cell cultures before they reach a mature state. This predicts that the onset of inhibition in the cultures must be strong with a connectivity much larger than in the end. Also the time-course of reaching firing rate homeostasis appears to be shorter than the curbing of this overly strong inhibition which takes longer and thus leads to the subcritical state observed in the interim. These are model predictions, because there is currently no data existing about the temporal development of inhibition. This data would be required to extend our model by implementing some time-course (dynamic coupling function) of the inhibitory development. Due to the lack of this data, this seems not useful at the moment because there is no way to constrain such a model extension. In general, the average homeostatic firing rate is independent of the level of inhibition and will in Phase III be reached regardless. All these predictions could be tested by measuring the degree of inhibition in the developing cultures and by pharmacologically interfering with the normal development forcing cultures to develop stronger (or weaker) inhibitory networks.
- Average rate 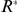 and membrane potential 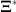 are at the end of the development fully invariant against system parameters and initial conditions with which development had started. Connectivity 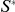 within the network, however, is influenced by the level of inhibition. Strong inhibition leads to more and weak inhibition to fewer connections. The latter could be assessed in parallel with the first prediction performing histological analyzes.
- For a network that has reached homeostasis, criticality can probably still be influenced by a sudden, prolonged change of inhibition. Following the second prediction, we expect some lasting connectivity changes to take place in these cases leading to a mildly changed criticality. Remarkably, first experimental evidence exists that an acute change in inhibition in fact alters criticality in mature cultured networks [1]. It further remains to be tested whether the mean firing rate will not be affected by a change in inhibition. It should quickly relax to its old value. Another recent experiment from Shew et al. (2009) [32] shows that AP5-DNQX, which blocks excitation, acts – at a first glance – like increasing inhibition in our model. However, it is not clear whether this experimental result and the model predictions can be compared at this stage, because theory suggests that inhibition acts dissipating in branching processes [33]. Thus, with respect to criticality decrease of excitation does not necessarily correspond to increase in inhibition. Nevertheless, it would be of great interest to assess whether this prediction holds and especially how long a homeostatic culture will still remain susceptible to such interference. Additional long term mechanisms, not captured by our model, might in reality terminate this effect after some time.
- The model further predicts that the actual balance between axonal supply 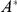 and dendritic acceptance 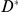 is quite sensitively depending on the initial conditions under which a cell culture starts its individual development. Thus, any histological analysis of connectivity 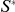 should best be performed by carefully assessing dendritic and axonal parameters in the different cultures. Even with very similar initial conditions, we expect those to vary widely across cultures, while the total connectivity 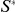 should be very similar. In this study this is reflected in the behavior of the PKC (Protein Kinase C)-inhibited cultures, which do not show any visible differences in their avalanche and firing rate characteristics as compared to non-treated cultures. The PKC-inhibited cultures express much richer dendritic structures [34]–[36]. However, the lack of difference in activity patterns found here suggests that the final synaptic density in these cultures does not substantially differ from that in the controls. The model predicts that this should go hand in hand with a shift of the fixed point along the hyperbola in Figure 8 C, which, however, does not alter the found avalanches. The prediction of a fix-point shift could be tested by detailed and complex histological analyzes of the existing synapses in controls and PKC-inhibited cultures, which goes beyond the scope of this study.

These predictions are quite specific as they do not depend on the parameter choices in the model, which is one strength of this approach. Most predictions, if not all, can be tested in a straight forward way in future experiments, albeit requiring substantial and sometimes difficult experimental work which can only be addressed in future work.

## Discussion

In the current study we have investigated how the activity patterns in developing cell cultures can be measured and modeled in terms of self-organized criticality. We have shown that the activity distributions in real cultures undergo a transition from a stage with little activity to a supercritical and then a subcritical state and finally to critical behavior. These transitions were significant for the cell cultures analyzed.

We used an extended version of the neurite outgrowth model by Van Ooyen and co-workers [10], [24], [25] with separate axonal and dendritic fields. The axonal and dendritic growth is driven by the goal to reach firing rate homeostasis as modeled in previous papers by Butz and co-workers [26], [27]. The model was able to reproduce the different developmental phases and several interesting predictions have been made.

### Relating criticality to morphological development

In general the chosen abstractions in the model appear to match the data description level quite well, but the question arises to what degree this still corresponds to reality in developing networks. Most importantly, the network stages described above must be related to the morphological development of dissociated neurons and their growing connectivity in culture which determines the activity pattern at every point in time [37]–[39].

It is well known [40] that the development of connectivity in cultures follows several phases. An initial phase (Phase I) is characterized by neuritic growth, followed (Phase II) by a structural overshoot and pruning followed by a maturation phase (Phase III) which finally leads to stable *mean* connectivity. Slowly growing connectivity in Phase I [41] leads over to the fast building of many synapses and a strong increase in activity in Phase II [38], [42], while pruning leads to Phase III with reduced number of synapses and lower activity [37]. Thereafter, firing behavior remains unchanged for two months [38], [42]. One may conclude that when synaptic pruning ceases, connectivity becomes stable and neuronal activities turn into homeostasis. Stable connectivity means that the sum of existing synapses does not vary much in time. The topology of the network can, however, not be predicted by the model as for this purpose a more detailed model of axons and dendrites would be required. However, neuronal development towards homeostasis substantially accelerates by increasing neuronal activities due to disinhibition by picrotoxin, a GABAergic synapse blocker [43]. Considering other transmitter studies, neuronal activity via increased glutamate release is likely to promote axonal outgrowth [44], [45] and therefore leads to a faster synapse formation and to an earlier maturation of the cell culture. Importantly, the behavior of dissociated neurons forming networks spontaneously occurs in any cell culture regardless of the original source of the plated neurons like cortex or hippocampus [46].

While certain simplifying assumptions had to be made to arrive at the basic differential equations (Equations 14–17) of our model, these experimental results clearly support the general dynamics assumed for our model.

In our model, networks with about 20% inhibition where the only ones that reached a robust critical state. While this level of inhibition corresponds to that in real nets, the results is intriguing as homeostasis of the firing rate will also be reached with much different levels of inhibitory cells. As known from literature [47], [48], GABA changes during the development from an excitatory to an inhibitory transmitter. As this is a fast process, inhibition sets in rapidly in the overshoot Phase II [48], [49] and possibly with a too high level. As discussed above, the observed subcritical phase clearly suggests a pruning phase for the inhibition which lasts longer than the firing rate equilibration. An indication of the functional role of synaptic pruning of inhibitory synapses was recently obtained from the developing auditory system in gerbils [31].

Like others [24], [25], [50], [51], also our model assumes that the main determining force within a growing network is the attempt of the neurons to achieve on average activity homeostasis. Several existing studies indicate that neurons, which are too active, seek to reduce their firing [50], [52], whereas neurons that are too quiescent try to increase it [53], [54]. Activity reduction is achieved by a reduction of the inputs to the cell (for example dendritic withdrawal) and vice versa. At the same time, highly activated cells respond with axonal outgrowth [44], [45], [55], [56] as increased levels of intracellular calcium, as a second messenger, regulates growth cone motility and therefore affects neurite outgrowth [44], [56]–[60].

### Self-organized criticality in neuronal networks

Self-organized criticality represents the situation that many systems of interconnected, nonlinear elements evolve over time into a critical state in which the probability distribution of avalanche sizes can be characterized by a power law. This process of evolution takes place without any external instructive signal. As analytically shown [7], an important feature of the power law is its scale invariance. This means that all neuronal avalanches regardless of their size (number of spikes) can be treated as physically equal [3]. Furthermore, avalanches remain stable in their spatial and temporal configuration for many hours, as already shown in cortical slices [15]. So, avalanches have optimal preconditions (equality and stability) to be a candidate for memory patterns. The stability of these effects is strongly supported by the way our model systems develop as will be discussed next.

The current study shows that networks in cell cultures undergo a certain transition during their morphological development. Thus, this paper is in the tradition of a sequence of investigations [17], [40], [43], [45] that try to link cell culture activity and development to possible in vivo stages. Indications exist indeed that different activity states in cultures could be matched to in vivo states [61], but one needs to clearly state that culture and in vivo development also show clear differences. In vivo development is much more structured which will lead to differences in (ongoing) activity. As discussed above, dendritic and axonal fine structure and their spatial distribution, however, does not seem to critically affect the observed state-transitions. Hence, this supports that, at the level of avalanches, little difference might indeed exist between culture and in vivo. A study by Stewart and Plenz [21] suggests that avalanche frequency is correlated to the integrated amplitude of local field potentials, which grows until 25 DIV in their study. This indicates that also their networks had developed from a low-activity state into states that follow a power-law distribution. They show that distributions have in general an exponent of −1.5, indicative of a branching parameter of 1 [1], and a closer look at their result suggests that transitory (sub- and supercritical) stages are also observable in this data set (see, e.g., Fig. 3D in Stewart and Plenz 2008 [21]). A related study by Pasquale et al. [17] confirms this observation. It, thus, seems that the critical state represents the final state of the development, which – in the model – is reached together with firing rate homeostasis. This leads to a high degree of stability, which would be desirable also from a functional viewpoint. This is supported by the observation that in Phase III in the model sudden changes of the network structure (e.g. by a sudden change of inhibition) will only lead transiently to a stronger disruption of criticality. Indeed, the system soon find its way back into homeostasis and criticality is only little affected.

Several previous studies [1], [17], [21], [32] focused on the exponent of the power law in the critical state. This is a characteristic parameter of the system and found to be around 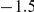 [1], [17], [21], [32]. We find that the exponent is 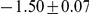 in simulations and 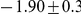 in cell cultures. Thus, the exponent matches previous results very well for the simulations. The difference in the experiments from the theoretical value of 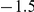 can occur from variations in the time bin, too harsh selection criteria, or a too small number of data points. Thus, deviations leading to the found value of 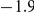 fall into the tolerance range of these experiments. In addition, it is not clear if the theoretical value of 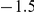 gained from branching processes [62] can be applied to all self-organizing systems in the critical state (f.e. Bak et al., 1987 [6]). Hence, it is equally well possible that the activity of the cultures is critical but does not exactly follow a branching process.

In a previous study Beggs and Plenz [1] have shown, that the critical state is optimal for a neuronal system concerning information flow. If the system is subcritical information will die out. The opposite situation is an epileptic system with too many long avalanches (supercritical state). Thus, a neuronal network in the critical state has the maximal dynamical range to react to incoming (external) information arriving from complex interactions of the neural system with its environment. The experimental part of the current study shows that real networks will develop towards such a state and the model suggests that this state is rather stable and therefore computationally reliable. Follow-up investigations, hopefully triggered by this research, might shed a light on the structural and functional dynamics of self-organized criticality in real developing brains and possibly also contribute a better understanding of developmental pathologies.

## Methods

### Experimental approach and data evaluation

#### Preparation of the cell cultures

Primary cortical cell cultures were prepared as described previously [63], [64]. Cells were derived from cortices of neonatal wistar rats by mechanical (chopping with scalpel, trituration) and enzymatical (0.05%, Trypsin, 15min at 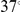
C.) dissociation and plated at densities (CASY cell counter, Innovatis) of 500,000 cells per 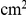 onto polyethyleneimine-coated micro-electrode arrays (59 TiN electrodes, 200/500
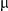
m electrode pitch, Multi Channel Systems). Cultures developed in 1ml growth medium, minimum essential medium (Gibco) supplemented with heat-inactivated horse serum (5%), L-glutamine (0.5mM), glucose (20mM) and gentamycin (10
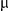
g/ml). One third of medium was exchanged twice per week. Cultures were maintained at 5% 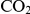 and 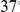
C. In a subset of cultures PKC (Protein Kinase C) was chronically inhibited by addition of a PKC antagonist (Gö6976, 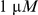, Calbiochem) at the first exchange of the culture medium at 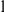 DIV. This different treatment has no significant influence on different parameters of the cell cultures (see Table 5) and, therefore, on the results of this paper.

**Table 5:** Cell cultures with PKC and without PKC (untreated) are compared.

	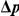	exponent	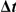 [ms]	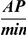	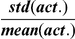	act. elec.
Untreated	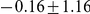	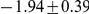	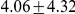	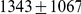	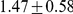	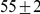
PKC	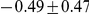	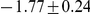	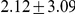	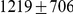	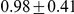	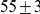

There are no significant differences. For the mean activity per minute the supercritical states are excluded as this state is too active leading to an unwanted bias in the data. For definition of the variables see the remainder of this Method section.

#### Electrophysiology

Electrophysiological recordings were performed on the different DIVs at the same time for one hour under culture conditions with a MEA1060-BC system amplifier (Multi Channel Systems) [65]. Raw electrode signals were digitally high-pass filtered at 200Hz and action potentials were detected by voltage threshold (3 times of standard deviation from the mean) using MC-Rack software (Multi Channel Systems).

#### Selection criteria

Clustering of neurons [66] at few electrodes can distort the avalanche statistics as clustering is a culture phenomenon and not seen in-vivo. To avoid clustering induced effects, we demand that activity has to be nearly uniformly distributed across all electrodes. So, all electrodes are excluded from the statistics which have measured more activity than two times the standard deviation from the mean activity per electrode. As shown in the literature [1], [3], [17] a too small number of measuring electrodes distorts the avalanche distribution (fewer long avalanches) and decreases classification of different states. This is avoided by choosing only samples with at least 50 active electrodes.

A total of 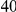 cultures has been originally considered in this study. Of the 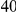 cultures 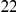 were controls and 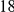 were PKC inhibited. A total of 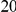 cultures did not obey the tight prior selection criteria for allowing rigorous criticality analysis and had to be excluded. The final number of analyzed cultures was, thus, 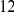 controls and 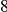 PKC inhibited. This shows that both groups were equally affected by the selection criteria. Interestingly, and also predicted by the model (see “Predictions of the Model”), no differences with respect to the activity analyzes of the current study were found between controls and PKC-inhibited cultures. Thus results were pooled.

#### Definition of avalanches

order to assess the distribution of avalanches in cell cultures and in the model in the same manner, we search for the beginning and the end of an avalanche by a gliding time bin of a fixed size. Whenever the system is silent (no spikes) for at least the duration of the time bin, an avalanche ends and a new one starts with the next spike. The time bin is the mean time interval between two spikes in the system. Too long time intervals are sorted out by first calculating the mean cross-correlation of all electrode signals and secondly by getting the time value for which 99% of the integration area is under this mean cross-correlation curve. This time value is the maximum time interval which is taken into account of the mean time interval [1]. This way we ensure that bursts on a longer timescales do not distort the statistics. This definition of avalanches can be used for cell culture and model data.

#### Measuring the deviation from a power law

To distinguish between the different states of an SOC system, the measure 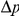 is defined. For this, the theoretical power law distribution 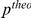 is calculated by a linear regression of the linear start on the left side (at approximately 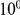) of the distribution 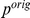 in the log-log-plot with MATLAB to the end of the linear behaviour. Now, for each data point 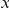 the regression 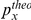 is subtracted from 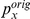. The mean of the differences of all data points is the measure 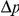.
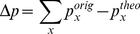
(11)We define the following relation between 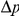 and the state of the system:
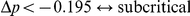


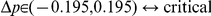


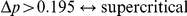



These thresholds are heuristic as there is no theoretical background for this. In general they correspond well to state classification if made by human inspection. Note, the results of this paper are not crucial dependent on narrow threshold margins.

#### Additional tests for criticality and data evaluation

**Figure 10:**
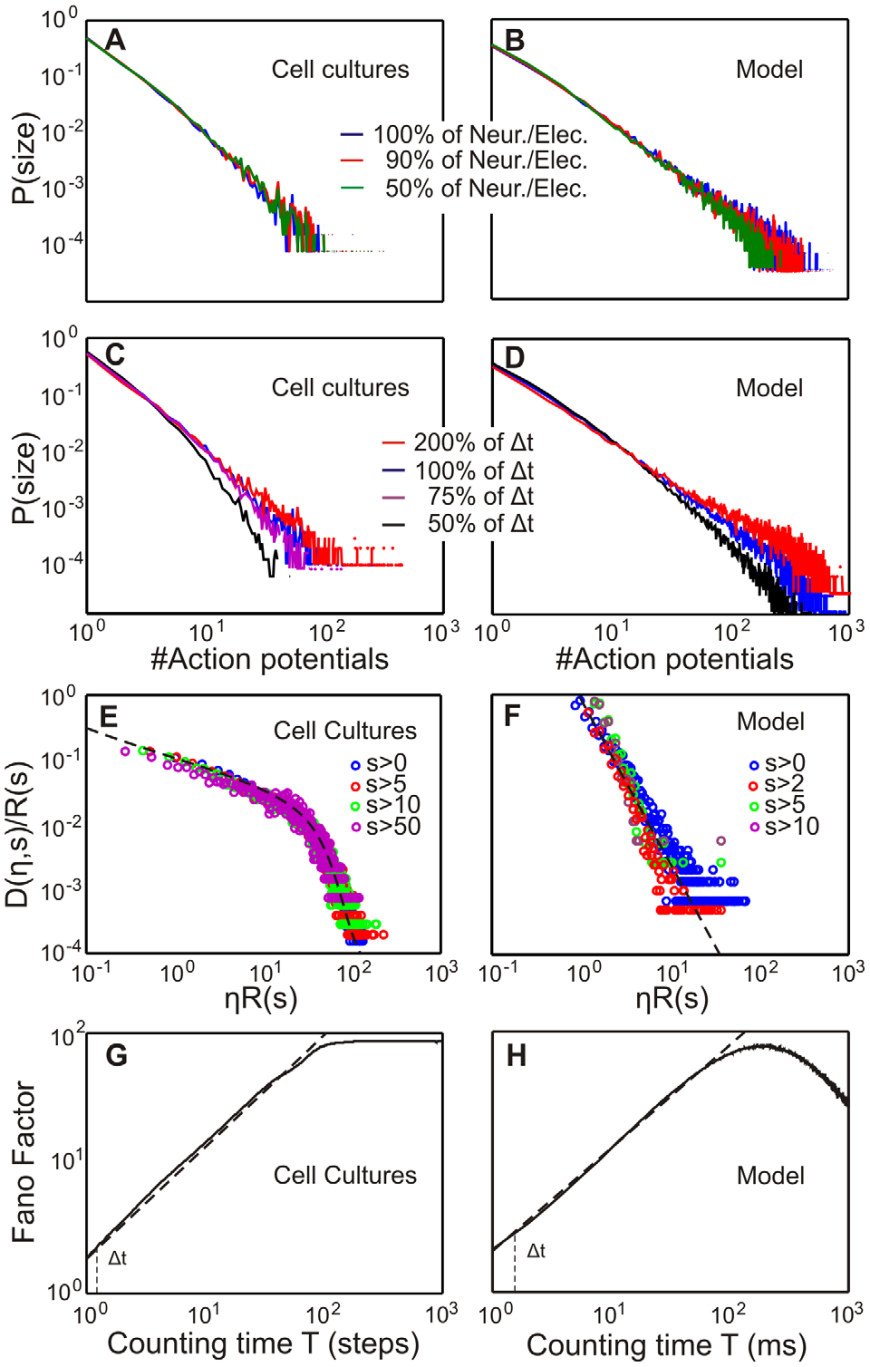
Additional tests for criticality used for cell cultures and model. In (**A,B**) we address potential spatial non-stationarity effects by comparing distributions obtained with only certain percentage subsets of the electrodes (neurons). In (**C,D**) we show that only minor variations exist for different time bins. Thus, temporal non-stationarities on a short time scale appear unlikely. Panels (**E,F**) show the scaling function 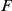 and, therefore, the scale-free behavior of model and cell cultures. Panels (**G,H**) show a Fano factor analysis for cell culture and model in the critical state. The exponent of the Fano Factor (linear regression) is 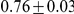 for the model and 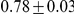 for cell cultures. Hence we conclude a scale-free clustering over different time scales 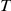.

Measuring a power law for the avalanche distribution is not sufficient to conclusively show that a system is in the critical state, because a power law can also result from the summation of two exponentials [7]. Therefore, the critical state in model and cell cultures has to be analysed by additional tests showing the scale-free behavior. Several tests were performed and results are shown here. First, the avalanche distribution has to show a power law even with less neurons (model) or electrodes (cell culture), indicating that the system is *spatially scale-free*. Figure 10 A,B demonstrates this. Furthermore, a system in the critical state has also to be *temporally scale-free*. To show this, different time bins are used for analyzing the avalanche distribution. Also these distributions show a power law relation (Figure 10 C,D) for model and cell cultures.

A third informative test is to assess the scale-free behavior of the inter-avalanche intervals (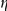). For this a minimum event size 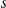 has to be introduced (minimum number of spikes in one avalanche) and then the time interval 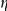 between the occurrence of two avalanches larger as 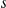 can be measured. Thus, for all values of 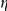 the probability distribution 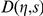 of getting an inter-avalanche interval 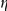 given 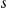 is assessed and can be re-scaled by the rate 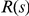 of having an avalanche larger than 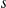 per time unit (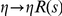, 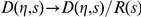). If this is done for different 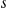 and all distributions form a single curve, the system is scale-free and the curve is the scaling function 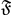 [67].
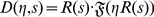
(12)This is done for our model and cell cultures in the critical state (Figure 10 E,F). Note, the actual shape of the different scaling functions is of less importance. The scale-free property is confirmed as long as all functions for a given system collapse onto the same function [67]. This is indeed observed in Figure 10 E,F.

Finally, a fourth test for criticality is the Fano Factor [68]–[70], for which the number of spikes 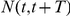 in a time window from 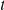 to 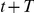 has to be considered using following equation
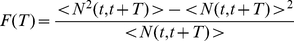
(13)The Fano Factor 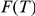 assumes a point process of events (spikes) and relates the clustering of these events to a Poisson process for which 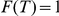. When the Fano Factor is below one, it indicates that the point process is more orderly than a Poisson process, and a Fano Factor above one indicates increased clustering at the given time scale 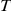 [69]. For a scale-free point process (e.g.; a system in the critical state), the Fano Factor needs to be a power law with the form 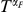. The exponent is an approximation of the 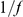 exponent which is related to the exponent of the avalanche distribution [9].

For the critical state in our model and cell cultures the Fano Factor shows a power law behavior for a wide range of time windows 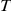 (Figure 10 G,H). There are no large differences between model and cell culture exponents (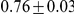 for model and 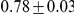 for cell cultures). Only at large values of 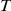, around 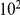 steps or 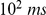, model and cell culture data do not show a power law behavior anymore and start to differ. However, the important range for the avalanche analysis is at smaller 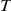-values (compare time bin 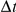 in Figure 10 G,H and Table 4).

### Computational modelling approach

In order to investigate the relationship between network development and self-organized criticality, we extended the previous neurite outgrowth model by Van Ooyen and Van Pelt [10], [24], [25] by separate axons and dendrites. The model is essentially a two-dimensional recurrent neuronal network with uni-directional synapses. Model neurons are described by four equations; for activity 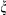, internal calcium concentration 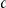 as well as dendritic acceptance 
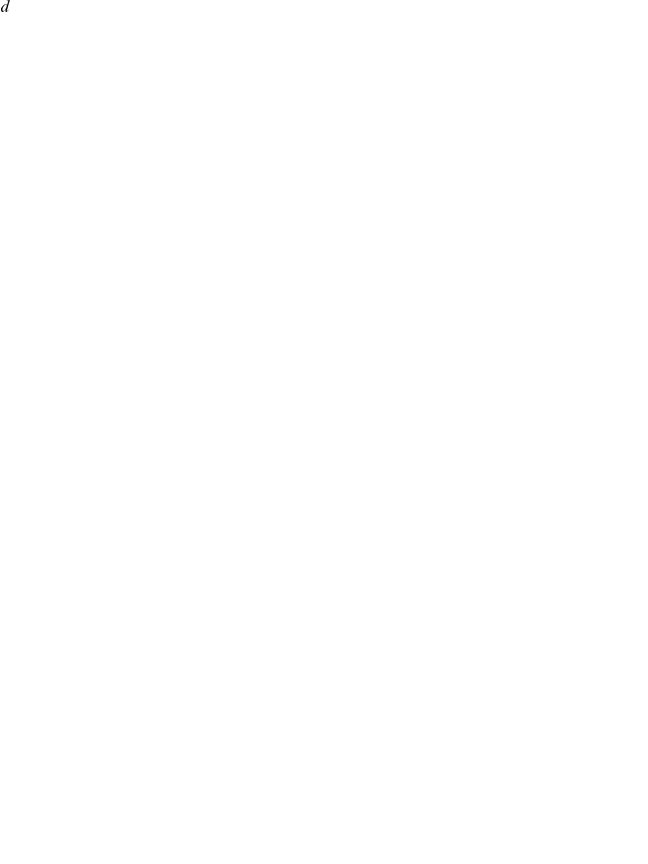
, and axonal supply 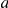. The last two parameters determine the connectivity 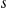 which is a generalisation of synaptic weights and the number of synapses between neurons. In line with previous experimental [27], [45], [50] and modelling studies [23], [24], [71], [72], the processes which determine the dynamics of this system can be summarized very briefly as: The activity of each neuron affects its calcium concentration. This, in turn, specifies the change of the dendritic and axonal offers, hence, the connectivity which will then gradually influence activity and so on. In the following we define parameters and equations. These equations are solved by the Euler method with an interval length of one simulated time step.

#### Membrane potential

As in the main text in the following, mean values are given as upper case letters, while lower case letters indicate individual values. For a fixed connectivity, given by the synaptic density between dendritic and axonal offers, each neuron has a certain activity. In accordance to the definition of the neuron model in the work by Abbott and Rohrkemper [10], the activity of the 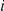-th neuron at the time point 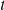 is given by a membrane potential 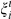, limited by a hard bound to 1, which decays in time exponentially with time constant 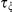 to 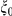, where 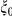 is the resting membrane potential. 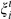 increases proportionally to the connectivity 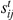 if a neighboring neuron 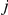 generates an action potential (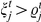; 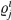 is a uniformly distributed number between 0 and 1. This relation between 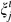 and 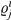 is obtained by the Heaviside-function 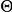).
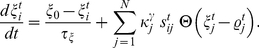
(14)

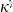
 defines if a presynaptic neuron is inhibitory (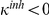) or excitatory (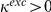). In the beginning of the simulation, all neurons are excitatory comparable to the very early development of biological neuronal networks [48]. At some point during simulation, a certain subset of neurons (20% of all) is converted into inhibitory neurons (see subsection *Phase II* of the *Results* section). We further define a refractory period of four time steps.

#### Calcium concentration

We model the calcium dynamics in our neuron model related to the work by Abbott and Rohrkemper [10]. The membrane potential 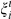 affects the calcium concentration 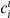 which has a slower exponential time constant 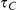. If a neuron 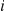 is active, it receives an influx of calcium and the concentration increases by 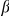.
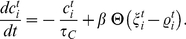
(15)

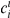
 determines the change of the synaptic density 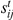.

#### Dendritic acceptance, axonal supply and connectivity

The development of dendrites and axons depends indirectly, by ways of the calcium concentration, on the activity. Lipton and Kater [45] showed that the deviation of the calcium concentration of a neuron from a certain target value determines its outgrowth. In line with previous modelling studies [10], [23], [24], [72], we defined a homeostatic value 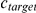 for which the axonal and dendritic offers (and with them the synaptic density) remains unchanged if 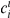 equals 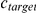. The rules for growth and retraction of continuous axonal supply and dendritic acceptance were taken from the modelling approach by Dammasch and Butz [23], [73] and can be described as follows: If the neuron has a too high membrane potential 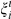, hence a too high calcium concentration 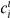, the dendritic acceptance 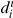 shrinks proportionally to a constant 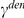 leading to a decrease of its input. If the membrane potential is too low, the dendritic acceptance increases to receive more inputs raising the calcium concentration by an increasing membrane potential to the homeostatic value. The axonal supply 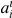 behaves equally but with inverted sign and different growth rate 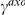.
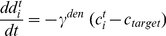
(16)

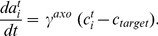
(17)Finally, we define the connectivity between neurons 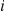 and 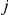 by:
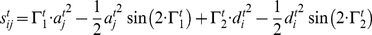
(18)with
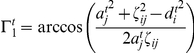


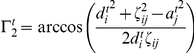
and 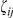 as distance between neuron 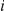 and 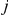. This essentially represents the overlap of the axonal and dendritic probability zones which can be understood as an abstract representation for the probability of synapse formation. In the simulations the neurons are distributed on a open grid to avoid developmental unsteadinesses followed from irregularities in the neuronal density. However, the overall behavior is not effected by this.

#### Parameter settings

The following table (**Table 6**) shows all standard parameter values. Exceptions are indicated in the text.

**Table 6:** Standard parameters used for the simulations.

Parameter	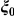	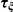	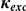	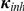	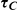	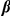	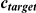	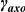	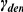
Value	0.0005	5	1000	see text	10	0.5	0.05	0.01	0.02

## Supporting Information

Text S1 Derivation of the steady state firing rate R^*^.
(0.03 MB PDF)

Text S2 Derivation of the nullcline of the model.
(0.03 MB PDF)
