# Supplementary material for: Self-Organized Criticality in Developing Neuronal Networks
Source: PLoS Comput Biol. 2010 Dec 2;6(12):e1001013. doi: 10.1371/journal.pcbi.1001013 (PMC2996321; doi:10.1371/journal.pcbi.1001013)
Supplement: Text S1 — Derivation of the steady state firing rate R*. (0.03 MB PDF) [file pcbi.1001013.s001.pdf]

Text S1: Derivation of the steady state firing rate $\mathbf{R}^*$

In the steady state (Phase III) we can assume that the calcium concentration of each neuron $c_i(t)$ is on average (measured over longer times) equal to c_{target}. Furthermore, we assume a flat distribution for the firing, hence averaging across avalanches and pauses. This way we can define $c_i(t)$ $\forall t \in (t_i^{sp}, t_i^{sp} + t_{max})$ via:

$$c_i(t) = c_i(t_i^{sp}) \cdot e^{-\frac{t-t_i^{sp}}{\tau_C}}, \tag{1}$$

where t_i^{sp} are the moments when a neuron fires and t_{max} the interval between two subsequent spikes. We get:

$$c_i(t_i^{sp}) = c_i(t_{max}) + \beta = c_i(t_i^{sp}) \cdot e^{-\frac{t_{max}}{\tau_C}} + \beta \tag{2}$$

$$\rightarrow c_i(t_i^{sp}) = \frac{\beta}{1 - e^{-\frac{t_{max}}{\tau_C}}} \tag{3}$$

$$\Rightarrow c_i(t) = \frac{\beta}{1 - e^{-\frac{t_{max}}{\tau_C}}} \cdot e^{-\frac{t-t_i^{sp}}{\tau_C}} \tag{4}$$

$$< c_i(t) > = \frac{1}{t_{max}} \int_{t_i^{sp}}^{t_i^{sp}+t_{max}} c_i(t)dt \tag{5}$$

$$= \frac{\beta}{t_{max}(1 - e^{-\frac{t_{max}}{\tau_C}})} \int_{t_i^{sp}}^{t_i^{sp}+t_{max}} e^{-\frac{t-t_i^{sp}}{\tau_C}} dt \tag{6}$$

$$= \frac{\beta \cdot \tau_C}{t_{max}(1 - e^{-\frac{t_{max}}{\tau_C}})} \left(1 - e^{-\frac{t_{max}}{\tau_C}}\right) \tag{7}$$

$$= \frac{\beta \cdot \tau_C}{t_{max}} \stackrel{!}{=} c_{target} \tag{8}$$

As average rate R^* is inverse to the average firing interval we finally arrive at:

$$R^* = \frac{c_{target}}{\beta \cdot \tau_C}. \tag{9}$$
