# Supplementary material for: Self-Organized Criticality in Developing Neuronal Networks
Source: PLoS Comput Biol. 2010 Dec 2;6(12):e1001013. doi: 10.1371/journal.pcbi.1001013 (PMC2996321; doi:10.1371/journal.pcbi.1001013)
Supplement: Text S2 — Derivation of the nullcline of the model. (0.03 MB PDF) [file pcbi.1001013.s002.pdf]

Text S2: Derivation of the nullcline of the model

We follow the approach of Van Ooyen and Van Pelt [1] and assume:

$$\frac{ds_{ij}^t}{dt} = 0, \qquad \forall i, j \in N \tag{1}$$

and

$$\frac{d\xi_i^t}{dt} = \frac{\xi_0 - \xi_i^t}{\tau_\xi} + \kappa \sum_{j=1}^{N} s_{ij}^t \ \Theta(\xi_j^t - \varrho_j^t) = 0, \quad \forall i, j \in N \tag{2}$$

Averaging over all neurons gets us:

$$0 = \frac{1}{N} \sum_{i=1}^{N} \left(\frac{\xi_0 - \xi_i^t}{\tau_\xi} + \kappa \sum_{j=1}^{N} s_{ij}^t \ \Theta(\xi_j^t - \varrho_j^t) \right). \tag{3}$$

Thus

$$0 = \frac{\xi_0}{\tau_\xi} - \frac{\Xi^t}{\tau_\xi} + \kappa \sum_{j=1}^{N} \ \Theta(\xi_j^t - \varrho_j^t) \ \frac{1}{N} \sum_{i=1}^{N} s_{ij}^t. \tag{4}$$

Now we replace the Heaviside function with a sigmoid as we can assume:

$$\Theta(\xi_j^t - \varrho_j^t) = \lim_{1/\alpha \to 0} \frac{1}{1 + \exp(\alpha(\varrho_j^t - \xi_j^t))}$$

$$\Rightarrow \quad \Theta(\xi_j^t - \varrho_j^t) \approx \frac{1}{1 + \exp(\alpha(\varrho_j^t - \xi_j^t))} = G(\xi_j^t)$$

On average we can set ϱ_j^t constant and equal to the mean value, which is 0.5 getting:

$$0 \approx \frac{\xi_0}{\tau_\xi} - \frac{\Xi}{\tau_\xi} + \kappa \sum_{j=1}^{N} \ F(\xi_j) \ \frac{1}{N} \sum_{i=1}^{N} s_{ij}. \tag{5}$$

Expanding G into a Taylor series we have:

$$G(\xi_j^t) \stackrel{\mathrm{T}}{=} G(\Xi^t) + G'(\Xi^t)(\xi_j^t - \Xi^t) + O^2(\Xi^t)$$

and get:

$$0 \approx \frac{\xi_0}{\tau_\xi} - \frac{\Xi^t}{\tau_\xi} + \kappa \sum_{j=1}^{N} \left(G(\Xi^t) + G'(\Xi^t)(\xi_j^t - \Xi^t) \right) \ \frac{1}{N} \sum_{i=1}^{N} s_{ij}^t. \tag{6}$$

$$0 \approx \frac{\xi_0}{\tau_\xi} - \frac{\Xi^t}{\tau_\xi} + \kappa \sum_{j=1}^{N} G(\Xi^t) \ \frac{1}{N} \sum_{i=1}^{N} s_{ij}^t \tag{7}$$

$$\approx \frac{\xi_0}{\tau_\xi} - \frac{\Xi^t}{\tau_\xi} + \kappa G(\Xi^t) \ \frac{1}{N} \sum_{j=1}^{N} \sum_{i=1}^{N} s_{ij}^t \tag{8}$$

$$\approx \frac{\xi_0}{\tau_\xi} - \frac{\Xi^t}{\tau_\xi} + \kappa G(\Xi^t) \ S^t \tag{9}$$

where we have used that one can write S^t as the average connectivity across all neurons, hence $S^t = \frac{1}{N} \sum_{j=1}^{N} \sum_{i=1}^{N} s_{ij}^t$, and get finally for the nullcline:

$$S^t = \frac{\Xi^t - \xi_0}{\tau_\xi \ \kappa \ G(\Xi^t)} \tag{10}$$

References

[1] Van Ooyen A, Van Pelt J (1994) Activity-dependent outgrowth of neurons and overshoot phenomena in developing neural networks. J Theor Biol 167: 27-43.
